# X-Linked Inhibitor of Apoptosis Protein – A Critical Death Resistance Regulator and Therapeutic Target for Personalized Cancer Therapy

**DOI:** 10.3389/fonc.2014.00197

**Published:** 2014-07-28

**Authors:** Petra Obexer, Michael J. Ausserlechner

**Affiliations:** ^1^Department of Pediatrics II, Medical University Innsbruck, Innsbruck, Austria; ^2^Tyrolean Cancer Research Institute, Innsbruck, Austria; ^3^Department of Pediatrics I, Medical University Innsbruck, Innsbruck, Austria

**Keywords:** SMAC-mimetic, nuclear factor kappa B, TAB1, BIR domain, tumor necrosis factor-alpha

## Abstract

Defects in apoptosis regulation are one main cause of cancer development and may result from overexpression of anti-apoptotic proteins such as inhibitor of apoptosis proteins (IAPs). IAPs are cell death regulators that, among other functions, bind caspases, and interfere with apoptotic signaling via death receptors or intrinsic cell death pathways. All IAPs share one to three common structures, the so called baculovirus-IAP-repeat (BIR)-domains that allow them to bind caspases and other proteins. X-linked inhibitor of apoptosis protein (XIAP) is the most potent and best-defined anti-apoptotic IAP family member that directly neutralizes caspase-9 *via* its BIR3 domain and the effector caspases-3 and -7 *via* its BIR2 domain. A natural inhibitor of XIAP is SMAC/Diablo, which is released from mitochondria in apoptotic cells and displaces bound caspases from the BIR2/BIR3 domains of XIAP thereby reactivating cell death execution. The central apoptosis-inhibitory function of XIAP and its overexpression in many different types of advanced cancers have led to significant efforts to identify therapeutics that neutralize its anti-apoptotic effect. Most of these drugs are chemical derivatives of the N-terminal part of SMAC/Diablo. These “SMAC-mimetics” either specifically induce apoptosis in cancer cells or act as drug-sensitizers. Several “SMAC-mimetics” are currently tested by the pharmaceutical industry in Phase I and Phase II trials. In this review, we will discuss recent advances in understanding the function of IAPs in normal and malignant cells and focus on approaches to specifically neutralize XIAP in cancer cells.

## The IAP Family – Structure and Function of IAPs

Aggressive cancer cells develop due to an accumulation of genetic and epigenetic abnormalities, defects in the intracellular signal transduction pathways, in proliferation and migration regulation, and the apoptotic cell death machinery. Cancer therapies are mainly designed to induce programmed cell death in highly proliferative tumor cells. When tumor cells acquire the ability to escape drug-induced cell death, either by defects in pro-apoptotic death regulators such as BH3-only proteins, or by overexpression of pro-survival proteins, these events will lead to the failure of chemotherapy. Such resistance mechanisms developed by single cancer cells result in the selection of chemotherapy- or radiation-resistant cancer cell subclones that finally will lead to therapy relapse. The inhibitor of apoptosis protein (IAP) member X-linked inhibitor of apoptosis protein (XIAP) is one culprit in the resistance to various apoptotic stimuli and frequently overexpressed in a number of different cancer types.

**Figure 1:**
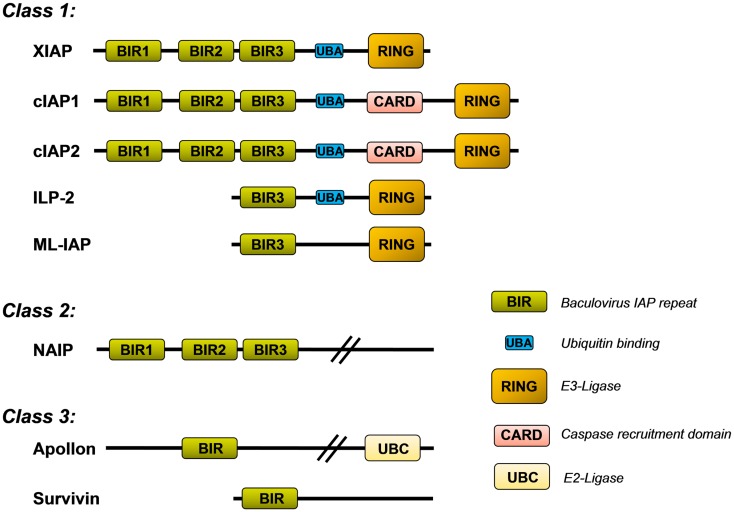
**Inhibitor of apoptosis protein (IAP) family**. Shown is the structure of the eight mammalian IAPs with their known functional domains (description in the main text).

The mammalian IAP family consists of eight different proteins that were originally described as apoptosis inhibitors as some of them can bind and neutralize caspases. They all share the so called baculovirus-IAP-repeat (BIR) domain and include neuronal IAP (NIAP/BIRC1), cellular IAP1 (cIAP1/BIRC2), cellular IAP2 (cIAP2/BIRC3), X-chromosome-linked IAP (XIAP/BIRC4), Survivin/BIRC5, BIR-containing ubiquitin-conjugating enzyme (BRUCE/Apollon/BIRC6), melanoma-IAP (ML-IAP/BIRC7), and finally IAP-like protein 2 (ILP-2/BIRC8). XIAP, cIAP1/2, ILP-2, and ML-IAP belong to the IAP-class 1 as they all contain a characteristical C-terminal RING (really interesting new gene) domain that acts as an E3-ubiquitin ligase (Figure 1). The IAPs also exert a number of additional functions in non-apoptotic pathways and contribute to, e.g., migration, invasion, and metastasis. In this family, XIAP is the only one that inhibits caspases by direct physical interaction (1). cIAP1 and cIAP2, for example, bind caspase-3 and -7, but do not efficiently inhibit them by physical interaction but mark them for proteasomal degradation (2).

X-linked inhibitor of apoptosis protein contains three BIR domains (BIR1–3) in the N-terminal half of the protein. Whereas BIR1 interacts with proteins that modulate NFkB signaling (3), BIR2 and BIR3 are critical for the interaction with either caspase-3 and -7 (BIR2) or caspase-9 (BIR3), respectively. The C-terminal part of XIAP carries a so called ubiquitin-associated (UBA) domain for ubiquitin-binding and a RING domain with E3-ubiquitin ligase activity, which is responsible for the recognition of protein substrates that are ubiquitinated by XIAP. This RING domain controls the stability of XIAP itself and also induces the proteasomal degradation of bound proteins such as caspase-3 (4) or the mitochondrial XIAP-inhibitor SMAC/Diablo (5).

## A Central Function of XIAP in the Regulation of Cell Death in Mammalian Cells

Apoptosis is a process initiated by a large number of signals that either activate specific membrane death receptors (extrinsic pathway) and/or intracellular pathways controlled by members of the Bcl2-family at the mitochondria (intrinsic pathway) (6). Both pathways converge at the level of specific proteases, called effector caspases that are the executioners of most forms of apoptosis. Fas ligand (FasL) and TRAIL, pro-apoptotic members of the TNF family, mediate their apoptotic signal via the “extrinsic pathway” by binding to their cognate receptors Fas/CD95 and TRAIL-R1 (DR4) or TRAIL-R2 (DR5), respectively. This induces the formation of a death-inducing signaling complex (DISC) that contains the adaptor molecule FADD and pro-caspase-8. As a consequence, autocatalytic cleavage of pro-caspase-8 and activation of a downstream caspase cascade occurs. In some cells, caspase-8 also connects to the mitochondria through cleavage of pro-apoptotic BID (type II cells) (7) and of the anti-apoptotic BCL2-protein MCL1_L_ (8), thereby providing a cross-talk between “extrinsic” and “intrinsic” death pathways.

**Figure 2:**
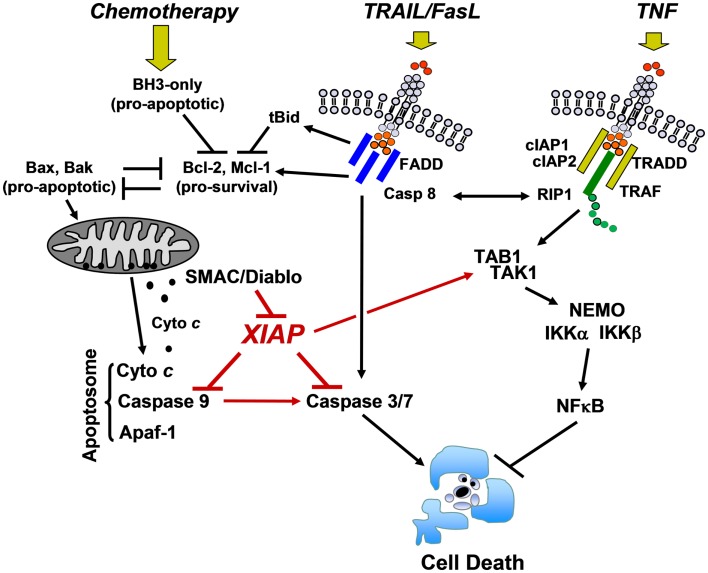
**Cell death/survival pathways controlled by XIAP**. DNA-damaging chemotherapeutics induce the expression of pro-apoptotic BCL2-proteins that antagonize the pro-survival function of BCL2, BclxL, and MCL1. Mitochondrial cell death is initiated by pore-formation *via* BAK and BAX, leading to the release of cytochrome *c* and other mitochondrial factors such as SMAC/Diablo into the cytoplasm. Cytochrome *c* triggers apoptosome formation and caspase-9 processing, which further activates downstream caspases-3 and -7. Death ligands (TRAIL and FasL) either activate executioner caspases-3 and -7 *via* caspase-8 directly or trigger mitochondrial cell death *via* caspase-8-mediated cleavage of BID and MCL1. XIAP physically interacts with caspase-9 at its BIR3 domain and with caspase-3 and -7 at its BIR2 domain and thereby interferes with both death signaling pathways. In contrast, TNF triggers a signaling cascade that, if RIP1 is ubiquitylated by cIAP1 and cIAP2, induces NFκB activation via TAK1/TAB1 and subsequent IKKα/IKKβ/NEMO complex activation. XIAP directly enhances this survival signal by forming a complex with TAB1/TAK1 via its BIR1 domain (description in the main text).

In this complex apoptosis signaling network mitochondria are central executioners of programmed cell death that integrate apoptotic signals, in particular in the “intrinsic pathway.” The “intrinsic pathway” is triggered by signals such as DNA damage, growth factor withdrawal, and anoikis. It is regulated at the level of mitochondria by the balance of pro- and anti-apoptotic BCL2-protein members (BCL2-rheostat). BCL2-family proteins control apoptosis primarily through the regulation of the mitochondrial outer membrane permeability (9, 10). Upon activation of the pro-apoptotic BCL2-proteins BAK and BAX which form pores in the outer mitochondrial membrane, cytochrome *c* and other mitochondrial proteins such as SMAC/Diablo and Omi/Htr are released from the mitochondrial inter-membrane space. Cytochrome *c* together with Apaf-1 forms the apoptosome complex that recruits pro-caspase-9, leading to caspase-9 processing and activation. The executioner caspases-3, -6, and -7 then degrade a plethora of cellular proteins and eventually also activate chromatin degradation via caspase-activated DNase (Figure 2). This opens the avenue for the coordinate fragmentation of cells into apoptotic bodies that can be recycled by neighboring cells.

**Figure 3:**
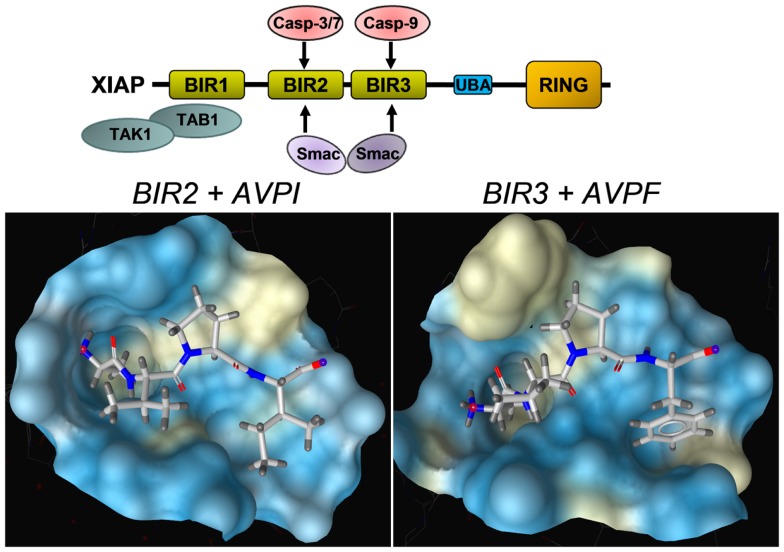
**Structural basis of the interaction between SMAC/Diablo and XIAP–BIR domains**. The amino acids alanine–valine–proline at the N-terminus of SMAC/Diablo interact with the binding groove present in the XIAP–BIR2 and the XIAP–BIR3 domain, but also with binding pockets in the BIR domains of a number of other IAPs. This explains why most SMAC-mimetics designed to neutralize XIAP also target other IAPs such as cIAP1 and cIAP2. Shown are crystal structures of the binding grooves of XIAP–BIR2 in complex with the peptide AVPI [PDB number: 4J64 (12)] and XIAP–BIR3 in complex with an AVPF peptide [PDB number: 2OPZ (13)]. Surfaces of binding pockets were calculated using LigandScout (Inte:ligand GmbH, Vienna). Color encoding represents aggregated lipophilicity/hydrophobicity (hydrophobic areas in yellow).

As mentioned above, XIAP interferes with these final steps in death execution as it binds partially processed initiator caspase-9 and the executioner caspases-3 and -7 via its BIR3 and BIR2 domains, respectively. The BIR3 domain of XIAP interacts with the Apaf-1/caspase-9 holoenzyme by sequestering the N-terminus of the small subunit of processed caspase-9. The N-terminal tetrapeptide of the processed caspase-9 that binds into the BIR3 pocket shares significant homology with the N-terminus of mitochondrial SMAC/Diablo, suggesting these two binding motives compete for XIAP–BIR3 interaction (11). SMAC/Diablo forms homodimers and the N-terminal ends of SMAC/Diablo-homodimers then bind to both, the BIR2 and BIR3 domain of XIAP and displace already processed caspases from the XIAP binding pockets. As shown in Figure 3, BIR2 and BIR3 both contain a deep, almost identical binding groove to anchor the N-terminal alanine–valine–proline amino acids of SMAC/Diablo (12, 13). By this process, SMAC/Diablo increases the amount of free, activated caspase-3, -7, and -9 and promotes the final steps of cell death execution. Another important mechanism of caspase inhibition by XIAP involves the E3 ligase activity of the RING domain. Schile et al. demonstrated that removal of the RING domain *in vivo* stabilizes the remaining XIAP protein but surprisingly also increases caspase-3 activity and TNF-sensitivity (14). This suggests that ubiquitylation of XIAP-bound caspases represents a second important mechanism for inhibition of caspase-mediated cell death. Thereby, high cellular levels of XIAP interfere with “extrinsic” as well as “intrinsic” death pathways and increase the resistance of cancer cells to various pro-apoptotic stimuli.

Similar to cIAP1, cIAP2, ML-IAP, and ILP-2, the RING domain of XIAP is capable of conjugating different types of ubiquitin chains to target proteins (15, 16). Protein ubiquitylation may lead to different physiological outcome, depending on whether one ubiquitin (monoubiquitylation) or multiple ubiquitin chains are linked to the substrate. In principle, eight different ubiquitin polymers can be generated. Each ubiquitin-linkage leads to different three-dimensional structures and serves different functions as they are differentially recognized by ubiquitin-associated (UBA) domains. Lys48-branched ubiquitin chains label proteins for proteasomal degradation, whereas Lys63 and Lys11 linear linkages serve as docking sites in signal transduction, endocytosis and other cellular responses such as DNA-repair [reviewed in Ref. (17)]. As shown in Figure 1, XIAP, like cIAP1, cIAP2, and ILP-2 contains such a UBA domain and thereby not only conjugates ubiquitin polymers to substrates but also recognizes highly polymerized ubiquitin chains and thereby functions as an ubiquitin receptor (18). This implies that the functions of IAP proteins are much more complex than simple protection against cell death by binding caspases. In the case of XIAP, the UBA domain is dispensable for caspase inhibition but necessary for the activation of the NFκB pathway by XIAP (18).

## XIAP-Induced Activation of NFκB Survival Signaling

The transcription factor NFκB plays a critical role in the development and malignant progression of cancer through its strong pro-survival functions. Due to the induction of different pro-survival proteins, such as Bfl1/A1 (19) or XIAP (20) by NFκB and its effects on angiogenesis, invasion, and metastasis of cancer cells, NFκB is one important culprit for the development of malignant diseases. This induction of resistance genes is in particular important in the case of radio-resistance, as ionizing irradiation also activates NFκB thereby contributing to both, DNA damage-induced apoptosis but also to a strong pro-survival signal in irradiated cells. DNA-double strand breaks induced by irradiation activate ATM, which in turn leads to the formation of a complex of p53-inducible death domain (PIDD)/RIP1 and NEMO (21) causing sumoylation and ubiquitylation of NEMO. This further activates the IKKα/IKKβ/NEMO signaling platform. This complex then phosphorylates IκBα, causing Lys48 ubiquitylation of IκBα and thereby, relieves NFκB from inhibition. It is believed that this strong NFκB-driven pro-survival signal during DNA-double strand breaks provides time to repair genotoxic stress-induced DNA damage.

One important upstream inducer of the NFκB pathway is the tumor necrosis factor alpha (TNFα)/TRADD/TRAF2 signaling complex (22) that is critically regulated by cIAPs (23, 24). TRAF2 directly interacts with cIAP1 (25, 26) and cIAP2 (27, 28) via the BIR1 domain, which leads to Lys11/Lys63 linear ubiquitylation of RIP1 (29–31) thereby generating a platform for the recruitment of TGFβ-activated kinase 1 (TAK1)/TAK1 binding protein 1 (TAB1), which promotes activation of the IKKα/IKKβ/NEMO signaling complex and thereby NFκB (32). Since TNFα is a transcriptional target of NFκB this signaling cascade constitutes an autoregulatory feed-back loop. In the absence of cIAP1/2 RIP1 is not ubiquitylated (24), which induces its interaction with the FADD/Caspase-8 complex and triggers apoptotic cell death (33).

The TAB1/TAK1 complex represents an important crosslink between NFκB signaling and XIAP: while the BIR2 and BIR3 domains of XIAP bind caspases, SMAC/Diablo or other substrates, the BIR1 domain directly interacts with the N-terminal domain of TAB1. Originally, it was thought that TAB1 is some kind of pseudophosphatase that regulates the accessibility of phosphorylated amino acids on TAK1 or downstream substrates and thereby modulates NFκB signaling via TAK1 (34). However, Lu et al. clearly demonstrated by crystallizing the TAB1/XIAP–BIR1 complex that the XIAP–BIR1 domain, for which no other function has been described until now, directly binds one molecule of TAB1 and thereby causes the formation of a complex where a dimer of XIAP-molecules binds two TAB1 proteins, which in turn recruit TAK1 proteins (3). This dimerization is important for TAK1 activation, as mutation of the BIR1 dimerization interface reduces the ability to activate NFκB. SMAC/Diablo does not directly interact with the BIR1 domain of XIAP, but interferes with XIAP–TAB1 interaction most likely by steric exclusion (3) and thereby impairs NFκB signaling *via* XIAP.

## XIAP Overexpression in Cancer

X-linked inhibitor of apoptosis protein is aberrantly expressed in a variety of human cancers and mediates resistance to chemotherapeutic drugs in specific subgroups of patients. High XIAP expression results in lack of therapy response leading to a change of therapy regimen and combined treatment with high dose radiotherapy. In cell lines isolated from acute myeloid leukemia (AML) XIAP overexpression is frequently observed: Tamm et al. investigated the expression of IAPs in 60 human cancer cell lines at mRNA and protein levels and found XIAP expressed in most malignant cells analyzed. The XIAP protein level correlated with the sensitivity to the anti-cancer drug cytarabine and other nucleosides (35). In childhood *de novo* AML XIAP overexpression is associated with an unfavorable response to induction chemotherapy and with a worse 3 year relapse free survival rate (36). Furthermore, XIAP shows maturation dependent expression differences and is associated with intermediate/poor cytogenetics in this childhood malignancy (37). In adult *de novo* AML aberrantly expressed XIAP is associated with monocytic differentiation in normal and malignant myelopoiesis, and also with the overall survival (38). In childhood acute lymphoblastic leukemia (ALL) the XIAP expression is highly increased by post-transcriptional regulation and is associated with poor *in vivo* glucocorticoid response and outcome. Resistance to glucocorticoid-induced apoptosis is one of the major risk factors for relapse and poor outcome in ALL (39). For childhood neuroblastoma, no clear clinical data is available about XIAP expression and clinical outcome, although XIAP-inhibitors significantly affect tumor growth and death resistance of neuroblastoma *in vitro* and *in vivo* (40, 41).

Furthermore increased XIAP levels have also been reported for ovarian carcinoma (42), B-cell Non-Hodgkin and Hodgkin lymphoma (43), clear cell renal cancer (44, 45), esophageal carcinoma (46), and non-small cell lung cancer (47). Chemoresistance after cisplatin treatment is linked to enhanced expression of XIAP in ovarian carcinoma cells (42). In clear cell renal cancer, the XIAP expression is an independent prognostic marker as it correlates with tumor aggressiveness. During clear cell renal cancer progression, an increase of XIAP relative to SMAC/Diablo occurs, which may contribute to apoptosis resistance (44, 45). Patients with renal cell carcinoma with low XIAP expression had a longer postoperative disease-specific survival as compared to those with high expression in the 5 year follow-up (48). Treatment of XIAP siRNA in combination with paclitaxel, cisplatin, fluorouracil, and etoposide enhanced chemosensitivity in esophageal carcinoma cell lines demonstrating that knockdown of XIAP may be a strategy for cancer therapy in patients with esophageal carcinoma (46). Also in non-small cell lung cancer, the high expression level of XIAP is involved in the pathogenesis of this cancer (47). In human prostate (49, 50) and hepatocellular carcinoma cells, the XIAP expression correlates with apoptosis resistance and increased metastatic foci *in vivo* (51). Patients with XIAP-positive hepatocellular carcinoma tumors showed a higher risk of relapse – in this tumor type XIAP can be defined as a biomarker that promotes metastasis and tumor recurrence (51).

The main challenge in the use of XIAP as a biomarker and therapeutic target is that, although XIAP is found overexpressed in many cancer tissues, its expression is not always correlated with adverse clinical outcome. This questions the relevance of XIAP as *the critical culprit* for therapy resistance in those malignancies, where elevated expression is observed. For example, although specifically elevated in non-small cell lung cancer (47), a recent study suggested no significant correlation between XIAP expression and patient outcome (52). In prostate cancer, XIAP is significantly higher expressed in the cancer tissue than in prostatic intraepithelial neoplasia, or in normal or benign hyperplasia, but, surprisingly, high expression of XIAP predicts lower risk of tumor recurrence than low or intermediate XIAP expression in the tumor tissue (53). This means that XIAP may play distinct roles in different types/subtypes of cancer and although it may serve as a biomarker for cancer tissue, targeting of XIAP in specific patient subgroups has to be carefully evaluated. In this respect, Xu and colleagues recently reported that significantly higher levels of XIAP are detected in breast cancer tissue *versus* normal breast tissue, but they could not confirm a correlation between XIAP expression, disease-free survival, and overall survival of breast cancer patients. However, when analyzing patient subgroups, they discovered that basal-like breast cancer patients with elevated XIAP expression had a significantly increased risk of tumor recurrence, suggesting that XIAP is predictive for poor relapse free survival in this patient subgroup (54). This suggests that additional clinical studies are required to identify those cancer patient subgroups that benefit from XIAP-targeted therapy. Therefore, XIAP in combination with other biomarkers may evolve as a predictive tool and as a drug target in personalized cancer therapy.

## Strategies to Interfere with Aberrantly Expressed XIAP in Cancer Cells

Patients that belong to subgroups where high cancer cell specific XIAP expression causes poor cancer therapy response may have a therapeutic benefit if they are treated with XIAP-targeting drugs that specifically neutralize the protective effect of XIAP. Such oncogene-specific neutralization might reduce the required doses of chemotherapeutics and thereby also lower therapy-related side effects. Therefore, XIAP is thought to be an excellent drug target for personalized cancer therapy.

In a classical approach by using antisense oligonucleotides (55), siRNA (56, 57) or morpholino-antisense (58), the decrease of the mRNA and protein levels of XIAP was shown to sensitize drug-resistant cancer cells to therapy-induced apoptosis or to induce even spontaneous cell death specifically in cancer cells. This is especially true for cancer cells with defective mitochondrial death signaling: repression of XIAP mRNA by RNAi or the specific XIAP-transcription-inhibitory compound Mithramycin A (59) overcomes TRAIL-resistance in carcinoma cells that show deregulation of the intrinsic apoptosis signaling pathway (60).

One such antisense strategy using the mixed backbone antisense oligonucleotides AEG35156 (Aegera Therapeutics) entered clinical trials, although several Phase I studies were terminated, one due to severe neurotoxicity (61). In a Phase I/II trial AEG35156 was effective in repressing XIAP mRNA levels and inducing apoptosis in CD34+CD38− AML stem cells. All Phase II patients showing AML stem cell apoptosis also achieved response (62). However, an open-label randomized Phase II trial of reinduction chemotherapy using a combination of high dose cytarabine and idarubicine therapy with and without the antisense oligonucleotides AEG35156 showed no improvement in the remission rates in patients with primary refractory AML although the therapy was well tolerated by the involved patients (63). As a consequence of these results, clinical studies on AEG35156 have been discontinued.

A second and even more promising approach is to sensitize cancer cells to chemotherapeutic drugs by functionally blocking XIAP via chemical compounds that bind into the BIR3 domains. The fact that SMAC/Diablo acts as a natural XIAP-antagonist and that the N-terminal ends of SMAC/Diablo bind with high affinity to the same binding pockets on the BIR2 and the BIR3 domain as caspases has led to the development of so called “SMAC-mimetics” that occupy the SMAC/Diablo-binding pockets in BIR domains of IAPs (Figure 3). These SMAC-mimetics are compounds structurally derived from the processed N-terminus of SMAC/Diablo and are in principle chemical derivatives of the peptide sequence AVPI (N-terminal part of SMAC/Diablo with the mitochondrial transit-sequence cleaved off). A plethora of different high affinity compounds have been developed to break the apoptosis block imposed by XIAP and other IAPs. However, some drawbacks also emerged with the development of these compounds that result from the complex cellular functions of their targets and from the fact that similar to the SMAC/Diablo protein almost all of these compounds target also the BIR domains of other IAPs, most prominently of cIAP1 and cIAP2, which leads to unwanted and in part toxic side effects. Whereas the genetic deletion of XIAP causes only a very mild phenotype in knock-out mice (64, 65), targeting of multiple IAPs, as it is achieved by high affinity SMAC-mimetics, may be detrimental to an organism as demonstrated by the deletion of cIAP1 together with cIAP2 or XIAP (66).

An important discovery for the understanding of SMAC-mimetic side effects, especially in combination therapies, was the observation that XIAP discriminates between type I and type II cells for Fas-induced apoptosis: in type I cells death receptor-induced activation of caspase-8 is sufficient to activate executioner caspases and thereby to induce apoptotic cell death independent of Bax and Bak. In type II cells, however, for full caspase-3 activation an amplification loop via mitochondria is needed. In these cells, mitochondrial outer membrane permeabilization is triggered by truncation of the BH3-only protein BID, which counteracts pro-survival proteins such as Bcl2 or BclxL, leads to Bax and Bak oligomerization, cytochrome *c* release, and caspase-9 activation at the apoptosome complex. Although type I cells (e.g., thymocytes) and type II cells (e.g., hepatocytes, pancreatic beta cells) contain similar steady state levels of XIAP, upon Fas binding to its cognate receptor, XIAP levels in type I cells are reduced, whereas XIAP concentration in type II cells increases. When XIAP is repressed or neutralized either by genetic ablation or by the use of the SMAC-mimetic BV6 (67) type II cells become sensitive to death receptor-induced cell death (68). This suggests that the activation of caspases in hepatocytes is normally blocked by XIAP and neutralization of XIAP may lead to significant damage of liver cells. In line with this study, Varfolomeev and colleagues found that BV6 in combination with FasL or DR5 agonistic antibodies rescues the effect of BID knockdown for apoptosis induction in different cancer cells (69).

Another important aspect of cell death regulation by IAP antagonists involves TNF-signaling and the discrimination between death induction via caspase-8 or survival signaling via stimulation of NF-κB signaling as outlined in Figure 2. In 2007, three independent papers reported different SMAC-mimetics, which induced caspase-8-dependent cell death when used as single agents (67, 70, 71). NF-κB stimulates the production of TNFα and activates the TNFα/TRADD/TRAF2 complex that associates with cIAP1 and cIAP2. cIAP1/2 ubiquitylate RIP1 (29–31) that in turn activates the NFκB pathway. SMAC-mimetic-induced inhibition of cIAP1 and cIAP2 results in auto-ubiquitination and proteasomal degradation of cIAP1/2 within minutes (67, 71), which further prevents ubiquitination of RIP1. As a consequence, RIP1 associates with the FADD/caspase-8 complex and triggers caspase-8-dependent cell death (33).

Due to these pleiotropic effects of SMAC-mimetics several drugs were designed that exert an improved activity against either XIAP or cIAP1/cIAP2. Ndubaku et al. described the structure-based design of SMAC-mimetics (compound C1–C3) that demonstrate high affinity to the BIR3 region of cIAP1 and cIAP2 (CS3: Ki cIAP1: 16 nM; Ki cIAP2: 85 nM) but only moderate affinity in micromolar range to the BIR3 domain of XIAP (Ki: >34 μM). Although, these compounds stimulated the NFκB pathway and induced cell death, these cIAP-selective compounds were significantly less effective than pan-SMAC-mimetics suggesting that for efficient cell death induction neutralization of XIAP and cIAP1/2 are required (72). To identify XIAP-specific SMAC-mimetics, XIAP-BIR2 domain specific small compounds have been designed that bind with low micromolar affinity to the XIAP-BIR2 domain and show significantly reduced Ki values for the BIR3 domain (73, 74). However, these authors did not provide data on the affinity to cIAP1/2. A recent report bei Kester et al. described the optimization of benzodiazepinones with high affinity (IC_50_ 45 nM) and high selectivity for the XIAP-BIR2 domain. These authors also provided evidence that the affinity of this compound to the cIAP1–BIR2 domain was about 500-fold less than to the XIAP–BIR2 domain (75). Although these are experimental drugs they are valuable tools to dissect the effects of targeting different domains on IAPs by small compounds in cancer cells.

**Figure 4:**
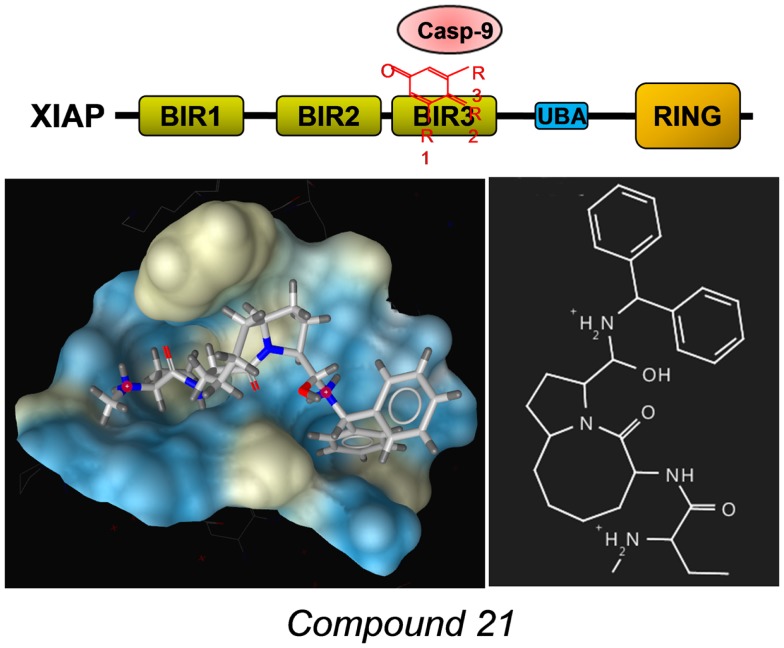
**Example for a SMAC-mimetic bound to the XIAP–BIR3 binding pocket**. Compound 21, a chemical derivative structurally related to the AVPI peptide was crystallized in complex with a XIAP–BIR3 protein fragment [PDB number: 2JK7 (79)]. From the calculation of the binding pockets, the similarity in the mode of binding between this SMAC-mimetic and the AVPF peptide (Figure 3) becomes evident. The surface of the binding pocket was calculated using LigandScout (Inte:ligand GmbH, Vienna). Color encoding shows hydrophobic areas in yellow.

A number of different SMAC-mimetics have demonstrated good anti-cancer activity in preclinical studies. On one hand, there are the monovalent SMAC-mimetics, small chemical compounds that occupy the BIR domains of XIAP and other IAPs and are able to sensitize cells to apoptotic stimuli or to induce apoptosis [reviewed in Ref. (76)]. Among the first compounds that bind as monomers to the BIR3 domains of IAPs were the synthetic mimetic SM-12d (77) and the natural compound embelin (78), both identified by the same group at the University of Michigan. The mode of binding of the mimetic *compound 21* was solved by the same group [PDB number 2JK7 (79)] also shown in Figure 4 with the surface of the binding pocket calculated using LigandScout software. The striking similarity between the AVPF peptide (Figure 3) and *compound 21* in the mode of binding to XIAP–BIR3 becomes evident when comparing binding pockets calculated on the basis of crystal structures. The orally active compound AT-406 (Ascenta Therapeutics), a further optimized chemical derivative of *compound 21* has reached clinical trials in the meantime (80).

Other compounds, e.g., small molecular XIAP-inhibitors from IDUN Pharmaceuticals/Pfizer were shown to cooperate with TRAIL to induce apoptosis and to inhibit clonogenic survival of childhood acute leukemia cells. These inhibitors kill leukemic blasts *ex vivo*. *In vivo*, they reduce leukemic burden in a mouse model of pediatric ALL engrafted in non-obese diabetic/severe combined immunodeficient mice (81). The authors also demonstrated that these inhibitors act synergistically with agonistic anti-CD95 antibodies or MegaFASL to induce apoptosis and reduce clonogenic survival in childhood acute leukemia cells (82). Additional early small-molecule XIAP-inhibitors such as the polyphenylurea-based antagonists 1396-12 and 1396-34 efficiently inhibited the growth of prostate cancer xenografts in mice and showed little toxicity on normal tissues (83). These polyphenylurea-based antagonists also exerted an antimetastatic activity against circulating metastatic prostate cancer cells (49).

However, only few of these compounds demonstrated sufficient affinity and specificity to be further developed into clinical studies. LCL-161, an orally active, monovalent SMAC-mimetic developed by Novartis also reached clinical studies and completed a Phase I multicenter trial for the treatment of solid tumors. Similar to LCL-161, the monovalent compound GDC-0152 developed by Genentech (84) has successfully completed a Phase I trial in patients with locally advanced or metastatic solid tumors (ClinicalTrials.gov).

A second class of SMAC-mimetics, the so called bivalent/dimeric mimetics consist in principle of two BIR-binding monovalent units and a bridging chemical linker. These dimeric compounds generally display a significantly higher affinity for IAPs than the monovalent mimetics and they are more effective in triggering apoptosis. Bivalent SMAC-mimetics such as SM-164 (85) simultaneously bind two BIR domains and despite their size also efficiently enter cells and are more potent than high affinity monovalent SMAC-mimetics in cell culture and animal experiments. Their disadvantage is that in contrast to monovalent XIAP-antagonists like LCL-161 or AT-406, which can be orally applied, these larger compounds require intravenous administration. Two of these compounds TL32711/Birinipant (Tetralogic Pharamceuticals) (86) and HGS1029 (Human Genome Sciences) also successfully completed Phase I trials on advanced ovarian, peritoneal cancer, other refractory solid tumors and lymphoma (TL32711), and advanced solid tumors (HGS1029) allowing the determination of the maximum tolerable dose (MTD).

Phase I studies assess the safety and tolerability of drugs to determine the MTD for further clinical trials. The fact that four SMAC-mimetics already passed this primary phase suggests that these compounds are tolerated. However, SMAC-mimetics are expected to act mainly also as chemosensitizing drugs and to this end all combination Phase I trials, e.g., AT-406 in combination with daunorubicine/cytarabine or TL32711 in combination with gemcitabine were terminated. Nonetheless, for all SMAC-mimetics that passed Phase I trials currently the recruitment for combination therapy Phase I trials has been launched. Phase II clinical trials testing the efficacy of the XIAP-antagonists have been set up in the case of LCL-161 for the treatment of triple negative breast cancer and forms of myelofibrosis as well as for TL32711 for the treatment of patients with acute myelogenous leukemia and ALL (ClinicalTrials.gov).

## Conclusion and Perspectives

Primary or acquired resistance of cancer to conventional chemo- and radiotherapy is the main cause for cancer-related death and remains a significant challenge in the therapy of malignant diseases. IAPs represent possible targets for specifically neutralizing therapy resistance mechanisms that lead to therapy failure and relapse. XIAP on one hand inhibits cell death execution by physical interaction with caspases and by causing their proteasomal degradation, on the other hand it also triggers survival signaling via the NFκB pathway thereby directly shifting the survival/death balance in cancer cells toward death resistance and NFκB-driven survival. The results from a plethora of preclinical studies, which demonstrate that XIAP-antagonists prove efficacy in enhancing drug- and radiotherapy-related cancer cell death and in specific cancer types even cause tumor remission are very promising. Therefore, in combination with diagnostic methods to identify patient subgroups that benefit from these novel compounds, XIAP-antagonists may be developed into highly efficient drugs in personalized medicine to overcome therapy resistance in cancer treatment. As several SMAC-mimetics have also been shown to neutralize circulating cancer cells, such drugs may also be useful in the clinics to prevent local recurrence of tumors after treatment as well as metastasis. Therefore, the targeting of IAPs in conventional cancer therapy has the potency to significantly improve success rates of cancer treatment and the survival of patients.

## Conflict of Interest Statement

The authors declare that the research was conducted in the absence of any commercial or financial relationships that could be construed as a potential conflict of interest.
